# Understanding the Influenza Vaccine Knowledge, Barriers, and Trust in the Hispanic Community of Metro Detroit

**DOI:** 10.7759/cureus.89068

**Published:** 2025-07-30

**Authors:** John Gallagher, Nevil Khurana, Rafael Ramos, Deni Peterson, Ingrid Rocha, Rene Andino-Galva, Alexander Eskandarian, Izabel Thomas, Rina Badran, Eliezer Gomez

**Affiliations:** 1 Family Medicine and Public Health Sciences, Wayne State University School of Medicine, Detroit, USA

**Keywords:** barriers to healthcare, hispanic health, influenza vaccine, metro detroit, vaccination attitudes, vaccination trust

## Abstract

Mistrust and barriers to influenza vaccination are well-documented in the literature. Underserved populations, including the Hispanic community, experience social determinants of health that exacerbate their health outcomes, including for common preventable illnesses such as influenza (flu). There is currently a gap in the literature regarding the knowledge, barriers to access, and trust of the Hispanic community regarding the influenza vaccine. The objective of this study was to assess knowledge, barriers to access, and trust regarding the influenza vaccine among Hispanic individuals in Metro Detroit. We administered a descriptive, mixed quantitative and qualitative survey, including predominantly Likert-style statements, to nearly 120 Hispanic individuals in Metro Detroit during the influenza seasons of 2023 and 2024 at our clinic and in public community spaces to assess these three domains. We also gathered information regarding demographics and previous vaccination history. These surveys were completed both via interviewer-administered and self-reported formats. For analysis, Likert-type items were collapsed, Chi-squared tests were performed, and the significance threshold was set at p <0.05. Findings included average knowledge of the vaccine, as 34% of individuals agreed they did not need the flu vaccine if they got it the prior year, while 44% of individuals disagreed. Trust in the vaccine was high, as 67% of respondents felt government influenza vaccination programs were trustworthy. Few overall barriers to vaccination were reported, as insurance was only reported by 19% as a barrier, while time was cited as the most common self-reported barrier by 51% of respondents. Subgroup analysis showed that, on average, males and younger individuals were less likely to trust influenza vaccines. Further research is needed to address time-related barriers and increase vaccine knowledge and trust through community initiatives. Such efforts will lead to improved vaccination rates and influenza outcomes for all Hispanic individuals.

## Introduction

Influenza (flu) is a serious infectious disease for which vaccination reduces both disease incidence and severity. Influenza incidence among United States (US) adults is approximately 5-9% annually, with a case fatality rate of about 0.1-0.2% [1,2]. Data from the US Centers for Disease Control and Prevention (CDC) show that Hispanic adults have higher rates of complications due to influenza than other ethnic groups, including 1.2 times higher rates of age-adjusted hospitalization than white adults from 2009 to 2023 [1]. CDC data shows that rates of influenza vaccination coverage in Hispanic adults are the lowest of all ethnic groups at 35% [3]. Given the relatively low rates of influenza vaccination in Hispanic adults, with relatively high rates of hospital admission and potential complications of influenza, increased influenza vaccine uptake in Hispanic individuals is needed and may lead to better outcomes in influenza incidence and complications in the Hispanic community.

However, multiple variables may influence influenza vaccine uptake in Hispanic adults. Previous research has shown that knowledge, trust, and barriers to access are principal factors that may affect vaccination rates in the US, including for the influenza vaccine. A recent study in North Dakota considered the concept of vaccine confidence as a multidimensional construct by which individuals form their opinions of vaccines through scientific knowledge, as well as their related trust in authority, vaccine products, and providers [4]. The authors concluded that providing accurate information was necessary to advance influenza vaccine knowledge and confidence in their target population [4]. Another study found that US adults were more likely to perceive influenza vaccines as effective and receive the vaccine if obtaining knowledge from medical sources, whereas they were less likely to exhibit such behaviors if receiving information from sources such as social media or family [5]. One other study showed that trust in the CDC by US adults was a strong precursor of influenza vaccination, and this effect was greater when insurance coverage and healthcare provider discussions were also present, emphasizing the importance of trust and access [6]. Research elsewhere, such as in Europe, showed that individuals who had high knowledge of vaccination were 26% more likely to receive vaccination during the past five years [7]. They also found that individuals were 10% less likely to receive vaccination if having to pay out of pocket, suggesting cost as a barrier [7]. Collectively, this research shows that variables such as knowledge, trust, and barriers to access may play a role in influenza vaccination. 

Studies have also investigated variables including knowledge, trust, and barriers to access regarding influenza vaccination in US Hispanic individuals. Based on a recent national survey, it was reported that Hispanics were the demographic group most likely to be the least aware and informed with respect to influenza vaccination [8]. Another study found that greater knowledge of influenza was positively associated with vaccination in Hispanic individuals in New York City [9]. The authors also found that believing the vaccine was not necessary or effective was often cited as the reason for not being vaccinated [9]. Another study found that trust or confidence in the influenza vaccine, particularly its safety, was associated with higher rates of vaccination in subgroups of Hispanic women of various national origins in Los Angeles [10]. One study found that Hispanic adults were more likely to have concerns about influenza vaccine side effects and report being vaccine-hesitant than non-Hispanic white adults [11]. Other research observed that barriers to access, such as living alone, lacking Medicare, financial strain, and difficulty accessing medical care, were strongly associated with decreased likelihood of influenza vaccination in Latinx individuals in Los Angeles [12]. These investigations have shown that variables such as knowledge, trust, and barriers to access affect influenza vaccination behaviors in Hispanic individuals in the US.

Some studies have sought to use community-based interventions to improve influenza vaccination behaviors in Hispanic communities. A community group in New York City disseminated information, gave presentations, and performed street-based and door-to-door vaccination to provide knowledge and gain trust while improving local vaccination rates of underserved Hispanic individuals [13]. One study in Washington used informational workshops and educational materials provided by community health workers to significantly improve knowledge and trust regarding influenza vaccines [14]. Such interventions may collectively address traditional barriers to vaccination and improve variables such as knowledge and trust to positively impact influenza vaccination rates in underserved Hispanic communities. In fact, in response to ethnic disparities in influenza disease severity and vaccination coverage, in 2022, the CDC called for such community-based interventions to improve influenza vaccination rates in underserved communities to improve influenza-related outcomes [3].

While previous research has suggested that knowledge, trust, and barriers may relate to influenza vaccination in US Hispanic individuals, no study has used a mixed methods survey to study all of these variables simultaneously. Moreover, many studies have focused on major metropolitan areas with large populations of Hispanic individuals, such as New York and Los Angeles [9,10,12,13]. The Hispanic population is among the fastest-growing in the US, including in Midwestern states such as Michigan. Data from the US Census showed Hispanic population growth in 81 of 83 Michigan counties between 2010 and 2020, particularly in the Detroit metropolitan area [15]. Regional data show that approximately 25% of Hispanic residents in Detroit are uninsured compared to 8.6% of non-Hispanic white individuals [16]. This insurance gap contributes to a significant barrier to healthcare access for preventative services, such as vaccination. However, other health data regarding the Detroit Hispanic community is scarce, including their unique knowledge, trust, and barriers regarding influenza vaccination. As community-based initiatives have effectively studied such variables in underserved Hispanic groups in other locations [13,14], such an effort may also apply to the underserved Hispanic community of Detroit.

The Amigos Medicos Clinic (AMC) is a student-run free clinic (SRFC) focused on preventative care in Southwest Detroit with an emphasis on primary care, patient education, health literacy, and vaccine initiatives. These efforts aim to improve knowledge, enhance trust, and address barriers to care, including for influenza vaccination. Currently, there is a gap in the literature describing the unique knowledge, barriers to vaccine access, and levels of trust of the Hispanic population of Detroit regarding influenza vaccination. Furthermore, the results of community-based initiatives for addressing this health need of the Hispanic community, particularly in major Midwest cities where the Hispanic population is growing, must be expanded. We have sought to use our community-based initiative to understand the knowledge, trust, and barriers regarding influenza vaccination amongst Hispanic residents of Metro Detroit.

This article was previously presented as a poster at the 2024 National Association of Free and Charitable Clinics Healthcare Symposium on September 30, 2024, the 2025 Latino Medical Student Association Midwest Regional Conference on February 15, 2025, and the Wayne State University School of Medicine 2025 Medical Student Research Symposium on March 7, 2025.

## Materials and methods

A cross-sectional, mixed quantitative and qualitative study was conducted to explore the knowledge, barriers to vaccine access, and levels of trust regarding the influenza (flu) vaccine and vaccines in general in the Hispanic community of Metro Detroit. Additionally, information regarding demographics and previous vaccination history was gathered. Data was collected through convenience sampling during AMC events and influenza vaccination clinics at a local community center in Southwest Detroit from September-November 2023, as well as in public spaces in Southwest Detroit during September-October 2024. This occurred through the use of a 23-question mixed quantitative and qualitative study, conducted in both interviewer-administered and self-administered formats, consisting of five major parts (demographics, vaccination history, knowledge, barriers, level of trust) in English and Spanish. Assistance was provided by interviewers to survey respondents for concerns related to technology and health literacy when appropriate.

Questions on the survey were adapted in part from the validated World Health Organization Strategic Advisory Group of Experts on Immunization Working Group on Vaccine Hesitancy Survey [17]. This resource covered topics related to vaccines, such as past experience, beliefs, knowledge, trust, and costs [17]. The first domain of our survey, demographics, asked basic questions about gender, age, ethnicity, and preferred language. These characteristics were self-identified by the respondents, including ethnicity. The second domain, vaccination history, asked general questions about vaccine behaviors, including influenza vaccination in the past five years, the same time frame used by Anastasiou and Heger in their 2021 study [7]. The third domain, knowledge, used Likert agree/disagree statements to cover different components related to the purpose and potential adverse effects of the influenza vaccine as covered in previous studies [9-11]. Barriers were assessed as the fourth domain by Likert statements covering previously reported barriers such as insurance and cost [6,7], as well as barriers potentially unique to our target community, including transportation and access/availability of the vaccine. Self-reported barriers were also included as a qualitative survey component. Finally, the fifth domain of trust was assessed through Likert statements regarding belief in vaccine efficacy, government programs, manufacturers, and ingredients as described in previous studies [4,5]. The items on our survey did not undergo additional validation or pilot testing but were partly informed by our previous survey-based research with our patient population through a health fair [18]. Potential respondents were offered an opportunity to complete the survey at their discretion and were presented with a research information sheet to explain the basis of the study. Upon their verbal consent and understanding, they began their participation in the study, with the ability to withdraw voluntary participation at any time. No protected health information was collected as part of this study, maintaining participants' anonymity. Study investigators were present in person with the participants at the time of survey completion to address any questions or concerns. A total of 120 survey responses were recorded.

Survey and procedure

The recruited participants were asked to complete a 23-question survey regarding their demographics, previous vaccination history, knowledge, barriers to access, and levels of trust regarding the influenza vaccine. The 23-question survey included yes/no questions regarding patient demographics (e.g., Do you identify as Hispanic or Latino?), multiple choice questions (MCQ) regarding previous vaccine history (e.g., How often in the past five years have you gotten the seasonal flu vaccine?), knowledge of the influenza vaccine using a Likert scale (e.g., If I am healthy, I don't need the flu vaccine), barriers to access using a Likert scale (e.g., It would be difficult for me to find transportation to make a flu vaccine appointment) in addition to qualitative self-reported barriers, and levels of trust regarding the influenza vaccine using a Likert scale (e.g., The flu vaccine manufacturers are trustworthy). Additionally, one optional free-response qualitative question was included asking the participants to explain their reasoning if they were unwilling to obtain the influenza vaccine. The surveys were available in both Spanish and English versions, which are included in the Supplemental Appendices for review. Study details were provided to participants, and verbal consent was obtained before their participation. The survey was completed in a mixed-mode format with both interviewer-administered and self-administered components. The investigators were present to assist respondents with concerns related to technology and health literacy.

A descriptive analysis was conducted to study the knowledge, barriers to access, and trust regarding influenza vaccines, in addition to the demographics and vaccination history of participants. For statistical analysis, Chi-squared was used. All comparisons were exploratory, not powered, and pre-specified. For Likert scale questions, collapse of categories was performed as follows: "Strongly agree" and "Somewhat agree" were collapsed to create one combined "Agree" category, while a "Disagree" category was created by collapsing of "Somewhat disagree" and "Strongly disagree" responses. Responses falling under "Neither agree nor disagree" were not included in the "Agree" or "Disagree" groups and were excluded from the Chi-squared analysis. Similarly, for other types of questions, responses were collapsed and analyzed by Chi-squared in categorical groups, according to categories of negativity ("Never" or "No") or positivity (including "Yes", "Sometimes", and "Once" or more). Chi-squared analysis was then performed to assess the significance of the proportions of survey respondents in agreement/positive categories compared to disagreement/negative categories. P-values were reported as significant if they were less than 0.05. Significant p-values indicated directionality in responses, with a statistically significant number of respondents indicating overall agreement/positive or disagreement/negative responses. Test statistics, including degrees of freedom and Cramer's V for categorical Chi-squared data were also calculated and interpreted. Conservative effect sizes for Cramer's V were used: a small effect size was considered 0.10, a medium effect size 0.30, and a large effect size 0.50.

Ethics statement

This study was approved as B3 Expedited/Exempt by our institution's Institutional Review Board, IRB #23-07-5982. The need for written informed consent was waived. All participants provided verbal informed consent prior to their participation, and no protected health information was collected.

## Results

The results of this study describe the knowledge, barriers to access, and trust regarding the influenza vaccine amongst the Hispanic community of Metro Detroit, as well as participant demographics and vaccination history. A total of 120 individuals responded to the survey, with 23 (98%) answering all questions. The most common age range selected was between 35-44 (34%), and 65% were between 35-64 (Table 1). Ninety-eight percent (117) of respondents identified as Hispanic or Latino/a, and 82% (97) stated that Spanish was their preferred language.

**Table 1:** Demographics of Survey Respondents

Demographic	Category	n (%)
Gender	Male	58 (49%)
	Female	61 (51%)
Age (years)	18-24	13 (11%)
	25-34	21 (18%)
	35-44	41 (34%)
	45-54	26 (22%)
	55-64	11 (9%)
	65+	7 (6%)
Hispanic or Latino/a	Yes	117 (98%)
	No	2 (2%)
Preferred language	Spanish	97 (82%)
	English	22 (18%)

The first portion of the survey assessed vaccination history and knowledge (Tables 2-7). Survey respondents were asked how often in the past five years they had gotten the seasonal flu vaccine. Twenty-nine percent (35) indicated that they received the vaccine every year. Thirty-four percent (40) of respondents indicated they get the vaccine "a few times" during this time period. Fifteen percent (18) of respondents marked that they received the vaccine once during the past five years. Twenty-two percent (26) of respondents had not obtained the vaccine once in the past five years. These responses were significant in directionality, with the majority of respondents in agreement (p<0.01, V=0.56). 

**Table 2:** Vaccination Beliefs of Survey Rrespondents

Question/Index	Yes	Sometimes	No	Total					Test Statistics			
If your doctor recommends a vaccine, do you usually get it?	80 (67%)	30 (25%)	9 (8%)	119					χ2(1)=85.72, p<0.01, V=0.85			
Do you think vaccines in general are necessary?	92 (77%)	25 (21%)	2 (2%)	119					χ2(1)=111.13, p<0.01, V=0.97			

**Table 3:** Vaccination History of Survey Respondents

Question/Index	Never	Once	A Few Times	Every Year	Total				Test Statistics			
How often in the past five years have you gotten the seasonal flu vaccine?	26 (22%)	18 (15%)	40 (34%)	35 (29%)	119				χ2(1)=37.72, p<0.01, V=0.56			

**Table 4:** Vaccination Knowledge of Survey Respondents

Index	Strongly Disagree	Somewhat Disagree	Neither Agree nor Disagree	Somewhat Agree	Strongly Agree	Total (n)	Test Statistics	
									The influenza vaccine may give me the flu.	29 (25%)	12 (10%)	23 (19%)	33 (28%)	21 (18%)	118	χ2(1)=1.78, p=0.18, V=0.14	
The influenza vaccine is safe for pregnant women.	16 (14%)	13 (11%)	42 (36%)	21 (18%)	26 (22%)	118	χ2(1)=4.26, p=0.04, V=0.24	
If I got the influenza vaccine last year, I don't need to get it again this year.	31 (26%)	21 (18%)	26 (22%)	21 (18%)	19 (16%)	118	χ2(1)=1.57, p=0.21, V=0.13	
If I am healthy, I don't need the influenza vaccine.	39 (33%)	23 (19%)	18 (15%)	17 (14%)	21 (18%)	118	χ2(1)=5.76, p=0.02, V=0.24	
The influenza vaccine may interact with medications.	29 (25%)	15 (13%)	44 (37%)	13 (11%)	17 (14%)	118	χ2(1)=2.65, p=0.10, V=0.19	

**Table 5:** Vaccination History by Age

Question/Index	Age	Never	Once, a Few Times, Every Year	Total	Test Statistics			
How often in the past five years have you gotten a seasonal flu vaccine?	18-44	21 (28%)	54 (72%)	75	χ2(1)=5.82, p=0.02, V=0.23			
	45-65+	3 (8%)	34 (92%)	37				

**Table 6:** Vaccination Behaviors by Age

Question/Index	Age	Yes	Sometimes	No	Total	Test Statistics		
Doctor recommends vaccine, usually get it?	18-44	43 (57%)	23 (31%)	9 (12%)	75	χ2=4.83, p=0.03, V=0.21		
	45-65+	32 (86%)	5 (14%)	0	37			

**Table 7:** Vaccination History and Knowledge by Gender

Question/Index	Gender	Strongly Disagree	Somewhat Disagree	Neither Agree nor Disagree	Somewhat Agree	Strongly Agree	Total	Test Statistics
									The influenza vaccine may give me the flu.	Female	14 (23%)	5 (8%)	13 (21%)	17 (28%)	12 (20%)	61	χ2(1)=0.505, p=0.48, V=0.07
Male	15 (26%)	7 (12%)	10 (18%)	16 (28%)	9 (16%)	57											
The influenza (flu) vaccine is safe for pregnant women.	Female	3 (5%)	4 (7%)	24 (39%)	15 (25%)	15 (25%)	61	χ2(1)=11.31, p<0.01, V=0.31
	Male	13 (23%)	9 (16%)	18 (32%)	6 (11%)	11 (19%)	57	
If I got the influenza (flu) vaccine last year, I don't need to get it again this year.	Female	17 (28%)	10 (16%)	16 (26%)	11 (18%)	7 (11%)	61	χ2(1)=0.43, p=0.51, V=0.06
	Male	14 (25%)	11 (19%)	10 (18%)	10 (18%)	12 (21%)	57	
If I am healthy, I don't need the influenza (flu) vaccine.	Female	23 (38%)	14 (23%)	9 (15%)	9 (15%)	6 (10%)	61	χ2(1)=3.85, p=0.04, V=0.18
	Male	16 (28%)	9 (16%)	9 (16%)	8 (14%)	15 (26%)	57	
The influenza (flu) vaccine may interact with medications.	Female	15 (25%)	6 (10%)	24 (39%)	9 (15%)	7 (11%)	61	χ2(1)=0.51, p=0.48, V=0.07
	Male	14 (25%)	9 (16%)	20 (35%)	4 (7%)	10 (18%)	57	

When examining participant history and general opinions regarding vaccines, 67% (80) of participants stated that if their doctor recommended a vaccine, they would get it. Twenty-five percent (30) responded that they would sometimes get it. These responses were also significant in directionality, with the majority of respondents in agreement (p<0.01, V=0.85). Additionally, 77% (92) responded that they do believe vaccines are necessary, and 21% (25) responded that they believe they are sometimes necessary. These responses were significant in directionality, with the majority of respondents in agreement (p<0.01, V=0.97).

The next portion of the survey attempted to identify gaps in knowledge regarding the influenza vaccine. Participants were asked if they believe the influenza vaccine may give them the flu. Forty-six percent (54) reported they strongly agree or somewhat agree, 35% (41) reported they strongly disagree or somewhat disagree, and 19% (23) reported they neither agree nor disagree. These responses were not significant in terms of directionality of participants agreeing or disagreeing (p=0.18, V=0.14). While not statistically significant, this finding is clinically significant, as it represents an area for potential vaccine education and literacy to differentiate between a "cold" and the "flu", as well as different types of vaccine (live versus attenuated), which may have different adverse effects. Thirty-two percent (38) of participants strongly agreed or somewhat agreed with the statement "If I am healthy, I don't need the influenza vaccine". Fifty-two percent (62) reported they either strongly disagree or somewhat disagree, and 15% (18) reported they neither agree nor disagree. These responses were significant in directionality, with the majority of respondents disagreeing (p=0.02, V=0.24). This finding is statistically and clinically significant since even healthy individuals may contract influenza and experience serious complications [19,20]. Thirty-four percent (40) of respondents strongly agreed or somewhat agreed with the statement "If I got the influenza (flu) vaccine last year, I don't need to get it again this year". Forty-four percent (52) either strongly disagreed or somewhat disagreed, and 22% (26) neither agreed nor disagreed. These responses were not significant in terms of directionality-participants agreeing or disagreeing (p=0.21, V=0.13). While not statistically significant, this finding is clinically significant, as annual vaccination is considered the mainstay of influenza prevention due to viral antigenic evolution [19,20]. Additional education to inform individuals about why annual influenza vaccination is necessary should be considered in a clinical setting. Forty percent (47) of participants either strongly agreed or somewhat agreed with the statement "The influenza vaccine is safe for pregnant women". Twenty-five percent (29) reported they either strongly disagree or somewhat disagree, and 36% (42) reported they neither agree nor disagree. These responses were significant in terms of directionality, with the majority of respondents agreeing (p=0.04, V=0.24). Apart from statistical significance, this finding is also clinically significant, as Hispanic women have high birth rates as compared to other ethnic groups, and influenza during pregnancy can lead to increased risk of maternal morbidity and death, as well as preterm birth weight and death in infants [21]. When asked if they believe the influenza vaccine may interact with their medications, 25% (30) reported they strongly agree or somewhat agree, 38% (44) reported they strongly disagree or somewhat disagree, and 37% (44) neither agreed nor disagreed. These responses were not significant in terms of directionality-respondents agreeing or disagreeing (p=0.10, V=0.19).

**Figure 1:**
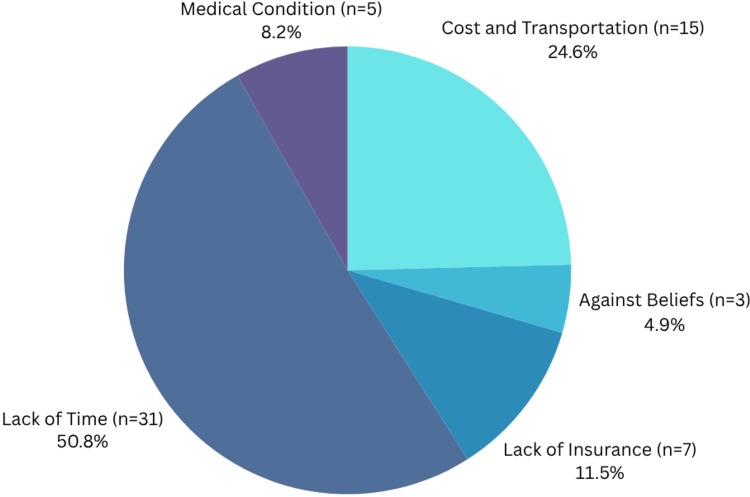
Reported Barriers to Vaccine Access

Possible barriers to vaccine access experienced by the participants were subsequently assessed (Table 8, Figure 1). Nineteen percent (23) strongly agreed or somewhat agreed with the statement "I need medical insurance to get the influenza (flu) vaccine", with 61% (71) either strongly disagreeing or somewhat disagreeing, and 20% (23) reporting they neither agree nor disagree. These responses were significant in terms of directionality, with the majority of respondents disagreeing (p<0.01, V=0.46). Twenty-four percent (28) indicated they strongly agree or somewhat agree that it would be difficult for them to cover the cost required for an influenza vaccine. Fifty-nine percent (69) strongly disagreed or somewhat disagreed with this statement, and 17% (20) indicated they neither agree nor disagree. These responses were significant in terms of directionality, with the majority of respondents disagreeing (p<0.01, V=0.38). Twenty percent (24) strongly agreed or somewhat agreed that it would be difficult for them to find transportation to make a flu vaccine appointment. Sixty-two percent (73) strongly disagreed or somewhat disagreed with this statement, and 17% (20) neither agreed nor disagreed. These responses were significant in terms of directionality, with the majority of respondents disagreeing (p<0.01, V=0.46). Twenty-four percent (28) of participants strongly agreed or somewhat agreed that they would not know where to get the flu vaccine if they wanted one. Fifty-seven percent (66) reported they strongly disagree or somewhat disagree with this, and 20% (23) neither agreed nor disagreed. These responses were significant in terms of directionality, with the majority of respondents disagreeing (p<0.01, V=0.36). Collectively, the responses in Table 8 show that, although previous data regarding insurance and access to healthcare in Hispanic individuals of Detroit shows many barriers to care [16], factors such as insurance, cost, transportation, and knowledge of vaccine availability are not considerable barriers to influenza vaccination for our surveyed respondents; results for each of these potential barriers were statistically significant with individuals in disagreement that they were barriers. Figure 1 shows self-reported participant responses when asked what barriers they experienced when getting the influenza vaccine. Respondents were allowed to report multiple barriers. "Lack of time" (51%, 31) was the most reported answer. These findings are clinically significant, as they reveal time constraints as the greatest obstacle for Hispanic individuals in Detroit to obtain influenza vaccination. This may relate to the many time-consuming jobs and roles these individuals already have as parents, workers, and/or students, an observation which may be clinically relevant to similar communities elsewhere.

**Table 8:** Perceived Barriers by Respondents

Index	Strongly Disagree	Somewhat Disagree	Neither Agree nor Disagree	Somewhat Agree	Strongly Agree	Total	Test Statistics		
I need medical insurance to get the influenza (flu) vaccine.	58 (50%)	13 (11%)	23 (20%)	12 (10%)	11 (9%)	117	χ2(1)=24.51, p<0.01, V=0.46		
It would be difficult for me to cover the cost required for an influenza (flu) vaccine.	49 (42%)	20 (17%)	20 (17%)	14 (12%)	14 (12%)	117	χ2(1)=17.30, p<0.01, V=0.38		
It would be difficult for me to find transportation to make an influenza (flu) vaccination appointment.	54 (46%)	19 (16%)	20 (17%)	18 (15%)	6 (5%)	117	χ2(1)=24.75, p<0.01, V=0.46		
I would not know where to get the influenza (flu) vaccine if I wanted one.	50 (43%)	16 (14%)	23 (20%)	13 (11%)	15 (13%)	117	χ2(1)=15.36, p<0.01, V=0.36		

Finally, participants were asked about their beliefs, trust, and reasons for refusal regarding the influenza vaccine (see Tables 9-12). Forty-seven percent (55) indicated they strongly agree or somewhat agree with the statement "The influenza (flu) vaccine prevents people from getting sick". Twenty-seven percent (32) indicated they either strongly disagree or somewhat disagree, and 26% (30) reported they neither agree nor disagree. These responses were significant in terms of directionality, with the majority of respondents agreeing (p=0.01, V=0.23). The survey asked if the participants believed government influenza vaccine programs are trustworthy, and 67% (78) stated they strongly agree or somewhat agree with this, while 15% (18) of the participants strongly disagreed or somewhat disagreed, and 18% (21) neither agreed nor disagreed. These responses were significant in terms of directionality, with the majority of respondents agreeing (p<0.01, V=0.57). Fifty-seven percent (67) of participants strongly agreed or somewhat agreed with the statement "The influenza vaccine manufacturers are trustworthy". Nineteen percent (22) indicated they either strongly disagreed or somewhat disagreed, and 24% (28) neither agreed nor disagreed. These responses were significant in terms of directionality, with the majority of respondents agreeing (p<0.01, V=0.44). Additionally, 53% (62) of participants strongly agreed or somewhat agreed that the ingredients of the influenza vaccine are non-toxic. Twenty-two percent (25) either strongly disagreed or somewhat disagreed with this statement, and 26% (30) neither agreed nor disagreed. These responses were significant in terms of directionality, with the majority of respondents agreeing (p<0.01, V=0.37). Collectively, the responses in Table 9 showed that the surveyed respondents were trusting of influenza vaccines, a statistically and clinically significant finding when considering possible reasons for low influenza vaccination rates in Hispanic individuals. Such rates may instead reasonably be due to time constraints as reported in Figure 1, though more investigation is needed.

**Table 9:** Trust of Respondents in Vaccines

Index		Strongly Disagree	Somewhat Disagree	Neither Agree nor Disagree	Somewhat Agree	Strongly Agree	Total	Test Statistics
The influenza (flu) vaccine prevents people from getting sick.		17 (15%)	15 (13%)	30 (26%)	21 (18%)	34 (29%)	117	χ2(1)=6.08, p=0.01, V=0.23
Government influenza (flu) vaccination programs are trustworthy.		13 (11%)	5 (4%)	21 (18%)	27 (23%)	51 (44%)	117	χ2(1)=37.50, p<0.01, V=0.57
The influenza (flu) vaccine manufacturers are trustworthy.		13 (11%)	9 (8%)	28 (24%)	32 (27%)	35 (30%)	117	χ2(1)=22.75, p<0.01, V=0.44
The ingredients in the influenza (flu) vaccines are generally non-toxic.		16 (14%)	9 (8%)	30 (26%)	27 (23%)	35 (30%)	117	χ2(1)=15.74, p<0.01, V=0.37

**Table 10:** Trust by Gender of Respondents

Index	Gender Category	Strongly Disagree	Somewhat Disagree	Neither Agree nor Disagree	Somewhat Agree	Strongly Agree	Total	Test Statistics
									The influenza (flu) vaccine prevents people from getting sick.	Female	9 (15%)	4 (7%)	15 (25%)	15 (25%)	18 (30%)	61	χ2(1)=3.05, p=0.08, V=0.16
Male	8 (14%)	11 (20%)	15 (27%)	6 (11%)	16 (29%)	56											
Government influenza (flu) vaccination programs are trustworthy.	Female	4 (7%)	2 (3%)	11 (18%)	19 (31%)	25 (41%)	61	χ2(1)=3.12, p=0.08, V=0.16
	Male	9 (16%)	3 (5%)	10 (18%)	8 (14%)	26 (46%)	56	
The influenza (flu) vaccine manufacturers are trustworthy.	Female	2 (3%)	4 (7%)	18 (30%)	20 (33%)	17 (28%)	61	χ2(1)=5.18, p=0.02, V=0.21
	Male	11 (20%)	5 (9%)	10 (18%)	12 (21%)	18 (32%)	56	
The ingredients in the influenza (flu) vaccines are generally non-toxic.	Female	3 (5%)	2 (3%)	22 (36%)	18 (30%)	16 (26%)	61	χ2(1)=8.74, p<0.01, V=0.27
	Male	13 (23%)	7 (13%)	8 (14%)	9 (16%)	19 (34%)	56	

**Table 11:** Trust by Age of Respondents

Index	Age Category	Strongly Disagree	Somewhat Disagree	Neither Agree nor Disagree	Somewhat Agree	Strongly Agree	Total	Test Statistics
									The influenza (flu) vaccine prevents people from getting sick.	18-44	13 (18%)	11 (15%)	19 (26%)	15 (20%)	16 (22%)	74	χ2(1)=3.02, p=0.08, V=0.16
45-65+	4 (9%)	4 (9%)	11 (26%)	6 (14%)	18 (42%)	43											
Government influenza (flu) vaccination programs are trustworthy.	18-44	9 (12%)	5 (7%)	17 (23%)	19 (26%)	24 (32%)	74	χ2(1)=3.11, p=0.08, V=0.16
	45-65+	4 (9%)	4 (9%)	0	8 (19%)	27 (63%)	43	
The influenza (flu) vaccine manufacturers are trustworthy.	18-44	11 (15%)	7 (9%)	19 (26%)	22 (30%)	15 (20%)	74	χ2(1)=6.43, p= 0.01, V=0.23
	45-65+	2 (5%)	2 (5%)	9 (21%)	10 (23%)	20 (47%)	43	
The ingredients in the influenza (flu) vaccines are generally non-toxic.	18-44	10 (14%)	5 (7%)	22 (30%)	19 (26%)	18 (24%)	74	χ2(1)=0.001, p= 0.98, V=0.0029
	45-65+	6 (14%)	4 (9%)	8 (19%)	8 (19%)	17 (40%)	43	

**Table 12:** Reasons for Vaccine Refusal

Index	Response							
If you are not willing to receive the influenza (flu) vaccine please indicate why:	"Creo que no es indispensable." (i.e., "I do not believe it is necessary.")							
	"I always get my vaccines."							
	"I don't know what's in it."							
	"He visto reacciones no buenas en algunos que se la han puesto." (i.e., "I have seen bad reactions in some who have gotten it.")							
	"No trust"							
	"No me interesa por el momento." (i.e., "I'm not interested at the moment.")							

Lastly, the association between previous influenza vaccination history and variables including vaccine attitudes, knowledge, trust, and barriers was also investigated (see Tables 13-18). Surveyed individuals who had been vaccinated against influenza at least once in the previous five years (included in the "V" group for "vaccinated") were compared to those who had not been vaccinated against influenza in the same time period (included in the "NV" group for "not vaccinated"). Significant or near-significant differences were noted for one question pertaining to vaccine attitudes/behaviors, regarding intention to get a vaccine if recommended by their doctor (p=0.01, V=0.39); one question related to vaccine knowledge, regarding need to get the influenza vaccine if received the previous year (p=0.05, V=0.18); and two questions regarding trust, namely trust in government influenza vaccination programs (p=0.04, V=0.19) and vaccine ingredients (p=0.06, V=0.18). For all of these items, individuals who had not been vaccinated against the flu in the previous five years were more likely to lean in the direction of hesitancy. No significant or near-significant differences were noted between the NV and V groups for items related to barriers. Figure 1 shows time to be the most frequent self-reported barrier by all surveyed individuals. Table 18 briefly summarizes the reported barriers by vaccination status, again showing time as the most frequently reported barrier regardless of vaccination status (though the NV group also frequently reported "Against beliefs" as a barrier to vaccination). 

**Table 13:** Vaccination Status by Gender

Demographic Gategory	Flu vaccination status (not vaccinated or vaccinated ≥ 1 time in past 5 years, NV or V)	Male	Female	Total	Test Statistics
Gender	Not vaccinated (NV)	14 (54)	12 (46)	26 (22)	χ2(1)=0.35, p=0.56, V=0.054
	Vaccinated (V)	44 (46)	49 (54)	93 (78)	

**Table 14:** Vaccination Attitudes by Vaccination Status

Index	Flu vaccination status	Yes	Sometimes	No	Total	Test Statistics
If your doctor recommends a vaccine, do you usually get it?	NV	11 (42)	8 (31)	7 (27)	26	χ2(1)=17.84, p=0.01, V=0.39
	V	69 (74)	22 (24)	2 (2)	93	
Do you think vaccines in general are necessary?	NV	15 (58)	10 (38)	1 (4)	26	χ2(1)=0.94, p=0.33, V=0.053
	V	77 (83)	15 (16)	1 (1)	93	

**Table 15:** Knowledge by Vaccination Status

Index	Flu Vaccination Status	Strongly Disagree	Somewhat Disagree	Neither Agree nor Disagree	Somewhat Agree	Strongly Agree	Total	Test Statistics
The influenza (flu) vaccine may give me the flu.	NV	5 (19)	1 (4)	6 (23)	7 (27)	7 (27)	26	χ2(1)=1.79, p=0.18, V=0.12
	V	24 (26)	11 (12)	17 (18)	26 (28)	14 (15)	92	
The influenza vaccine is safe for pregnant women.	NV	5 (19)	1 (4)	7 (27)	5 (19)	8 (31)	26	χ2(1)=0.47, p=0.50, V=0.063
	V	11 (12)	12 (13)	35 (38)	16 (17)	18 (20)	92	
If I got the influenza vaccine last year, I don't need to get it again this year.	NV	6 (23)	1 (4)	7 (27)	6 (23)	6 (23)	26	χ2(1)=3.77, p=0.052, V=0.18
	V	25 (27)	20 (22)	19 (21)	15 (16)	13 (14)	92	
If I am healthy, I don't need the influenza vaccine.	NV	6 (23)	5 (19)	5 (19)	4 (15)	6 (23)	26	χ2(1)=1.044, p=3.069, V=0.094
	V	33 (36)	18 (20)	13 (14)	13 (14)	15 (16)	92	
The influenza vaccine may interact with medications.	NV	6 (23)	3 (12)	11 (42)	3 (12)	3 (12)	26	χ2(1)=0.002, p=0.96, V=0.0041
	V	23 (25)	12 (13)	33 (36)	10 (11)	14 (15)	92	

**Table 16:** Barriers by Vaccination Status

Index	Flu Vaccination Status	Strongly Disagree	Somewhat Disagree	Neither Agree nor Disagree	Somewhat Agree	Strongly Agree	Total	Test Statistics
I need medical insurance to get the influenza (flu) vaccine.	NV	10 (38)	2 (8)	7 (27)	5 (19)	2 (8)	26	χ2(1)=1.97, p=0.16, V=0.13
	V	48 (53)	11 (12)	16 (18)	7 (8)	9 (10)	91	
It would be difficult for me to cover the cost required for an influenza (flu) vaccine.	NV	10 (38)	2 (8)	5 (19)	5 (19)	4 (15)	26	χ2(1)=2.56, p=0.11. V=0.15
	V	39 (43)	18 (20)	15 (16)	9 (10)	10 (11)	91	
It would be difficult for me to find transportation to make an influenza (flu) vaccination appointment.	NV	13 (50)	4 (15)	5 (19)	2 (8)	2 (8)	26	χ2(1)=0.47, p=0.50, V=0.063
	V	41 (45)	15 (16)	15 (16)	16 (18)	4 (4)	91	
I would not know where to get the influenza (flu) vaccine if I wanted one.	NV	11 (42)	2 (8)	7 (27)	4 (15)	2 (8)	26	χ2(1)=0.037, p=0.84, V=0.018
	V	39 (43)	14 (15)	16 (18)	9 (10)	13 (14)	91	

**Table 17:** Trust by Vaccination Status

Index	Flu Vaccination Status	Strongly Disagree	Somewhat Disagree	Neither Agree nor Disagree	Somewhat Agree	Strongly Agree	Total	Test Statistics
The influenza (flu) vaccine prevents people from getting sick.	NV	7 (27)	3 (12)	7 (27)	3 (12)	6 (23)	26	χ2(1)=2.63, p=0.11, V=0.15
	V	10 (11)	12 (13)	23 (25)	18 (20)	28 (31)	91	
Government influenza (flu) vaccination programs are trustworthy.	NV	7 (27)	0	6 (23)	6 (23)	7 (27)	26	χ2(1)=4.38, p=0.036, V=0.19
	V	6 (7)	5 (5)	15 (16)	21 (23)	44 (48)	91	
The influenza (flu) vaccine manufacturers are trustworthy.	NV	6 (23)	1 (4)	7 (27)	6 (23)	6 (23)	26	χ2(1)=1.91, p=0.16, V=0.13
	V	7 (8)	8 (9)	21 (23)	26 (29)	29 (32)	91	
The ingredients in the influenza (flu) vaccines are generally non-toxic.	NV	7 (27)	1 (4)	9 (35)	5 (19)	4 (15)	26	χ2(1)=3.60, p=0.058, V=0.18
	V	9 (10)	8 (9)	21 (23)	22 (24)	31 (34)	91	

**Table 18:** Self-Reported Barriers by Vaccination Status

Barriers to Influenza Vaccination	Not Vaccinated	Vaccinated
Against Beliefs	5 (31)	3 (5)
Lack of Insurance	2 (13)	7 (11)
Lack of Time	5 (31)	31 (51)
Medical Condition	0	5 (8)
Cost, Transportation, and Availability	4 (25)	15 (25)

## Discussion

Our study sought to describe the knowledge, barriers to access, and trust regarding influenza vaccination in a growing, yet understudied, Hispanic population of Metro Detroit. Additionally, we sought to characterize the demographic information and previous vaccination history of participants to better understand the unique patient population our clinic serves in Southwest Detroit. Our principal findings include an average knowledge base, fewer than expected barriers to access, and high levels of trust regarding the influenza vaccination. This is potentially clinically significant considering that previous studies had found that Hispanic individuals were the least informed regarding influenza vaccination [8], had many barriers to influenza vaccination [12] and healthcare [12,16], lower trust/confidence regarding the influenza vaccine [11,22,23], lower vaccination rates, and worse outcomes [3]. However, selection bias may have played a role in our study, as the surveyed individuals were chosen via convenience sampling at clinic events (suggesting health-seeking behavior) and public community spaces, introducing sampling bias and potentially limiting generalizability. Notably, we also found that Hispanic males and young individuals overall were less trusting of the influenza vaccine. Our study successfully reached the target population, with a sample size of 120 participants, 98% of respondents identifying as Hispanic or Latino/a, and 82% reporting Spanish as their preferred language.

While many previous reports have assessed barriers [10,12,13,15] and mistrust [14] regarding vaccines in vulnerable populations, few have considered knowledge regarding common vaccine concepts. Our study sought to assess the knowledge of the Metro Detroit Hispanic population related to some of these issues. Our findings yielded a greater than expected understanding of the mechanism, purpose, and adverse effects of vaccines. For instance, respondents agreed or felt indifferent (76%) that the influenza vaccine is safe for pregnant women (p=0.04, V=0.24), a finding which the Centers for Disease Control and Prevention have emphasized [18]. Additionally, most respondents (66%) understood or at least felt indifferent that the flu vaccination is an annual shot, recommended once per year (p=0.21, V=0.13). Fifty-two percent of respondents also understood that even if they are healthy, they may need the flu vaccine (p=0.02, V=0.24). Interestingly, respondents were split on whether the flu vaccine could give someone the flu, with 46% in agreement, 35% in disagreement, and 19% indifferent (p=0.18, V=0.14). Overall, these results show acceptable knowledge regarding several characteristics of the flu vaccine, including mechanism, purpose, and adverse effects, with some key areas for improvement.

Many previous findings also suggest that the Hispanic population disproportionately faces barriers to vaccination [10,12,13,15] and medical care [3,7,8,9], yet our findings suggest that, at least as it relates to flu vaccination, Hispanic individuals in Metro Detroit experience few access-related barriers to care. A majority of our participants (61%) do not feel they need medical insurance to get the vaccine (p<0.01, V=0.46). Similarly, 59% and 62% do not feel that cost or transportation are barriers for receiving the vaccine (p<0.01, V=0.38; p<0.01, V=0.46, respectively). Knowledge of where to get the vaccine (57%) is also quite high (p<0.01, V=0.36). Overwhelmingly, contrary to the suspected access-based barriers to vaccination, as depicted in Figure 1, respondents report that 'Lack of time" (31%) and "Other" (43%) are the most prevalent barriers. This possibly reflects the multi-occupational status of our survey respondents (83% aged 25-64), many of whom may be parents and/or full-time workers. Indeed, previous literature suggests that an "inconvenient time for vaccination" may play a role in individuals' reluctance to become vaccinated [19].

Our findings suggest that trust levels regarding vaccines are generally high in our sampled population, including in vaccine efficacy, government programs, manufacturers, and ingredients/composition. This differs from some studies, which showed low trust in influenza vaccines and efficacy among Hispanic individuals [11,22,23]. Our findings may relate to the health-seeking nature and the overall high proportion of previous vaccination in many of our survey respondents. Additionally, previous research showed that general trust in healthcare professionals increases vaccine uptake in Hispanic individuals and countries [24]. Universal vaccination efforts exist in Latin American countries such as Mexico [25], and many Hispanic individuals in Metro Detroit are of Mexican descent and/or foreign-born; thus, they may be generally more trusting of vaccines, as reflected in our results. Forty-seven percent of respondents agree that flu vaccines prevent people from getting sick (trust in vaccine efficacy, p=0.01, V=0.23), and 67% agree or strongly agree that government vaccine programs are trustworthy (trust in government, p<0.01, V=0.57), which was an unusually strong association by effect size. 57% of respondents agree or strongly agree that manufacturers are trustworthy (trust in production, p<0.01, V=0.44), and 53% agree or strongly agree that vaccine ingredients are generally non-toxic (trust in vaccine composition, p<0.01, V=0.37).

Notably, our study suggests that Hispanic males and young individuals may be less trusting of the influenza vaccine. It is documented that generally vaccination acceptance increases with age and female sex. One study examining the Big 5 personality model trait of agreeableness found that lower agreeableness is associated with vaccine hesitancy and uptake refusal [26]. Males have been well-established to score lower than females on this dimension of agreeableness [27], supporting our findings of vaccine hesitancy in Hispanic males. Thus, our findings that Hispanic males and young individuals (age 18-44) may be less trusting of the flu vaccine, including manufacturers (p=0.02, V=0.21; p=0.01; V=0.23, respectively), are of interest for future investigations and directed patient education efforts.

Limitations

While our study has a number of interesting findings, there are several limitations that we must recognize. First, a subset of our participants (25 respondents, 21% of the total) were AMC patients receiving medical care and/or the influenza vaccination at our clinic, suggesting a predisposition towards health-seeking behaviors and general vaccine acceptance. This may have introduced selection and healthy user bias, skewing the results of our study in favor of positive reporting of knowledge, barriers, and trust towards the influenza vaccine. To overcome this, we also included responses of patients in the community (95 respondents, 79% of the total), more representative of an average person who is neither inclined nor disinclined towards health-seeking behaviors. However, in both settings, convenience sampling was performed, possibly leading to selection and sampling bias, which may limit the generalizability of our results to the target population as a whole. Additionally, a large proportion of survey respondents (78%) had received the influenza vaccination at least once in the previous five years, possibly introducing sampling bias and skewing our results in favor of vaccination. However, only 29% of respondents had received the influenza vaccine every year, and 34% a few times during the previous five years, comparable to the 35% of Hispanic adults nationally who received the annual influenza vaccine in the 2023-2024 season per the CDC [1]. This suggests that our surveyed population had similar rates of influenza vaccination compared to the national average for Hispanic adults, supporting the generalizability of our findings. Unfortunately, we also encountered many individuals in the community who were either unwilling or unable to participate in our survey. Having their opinions included in our findings would have indicated a more diverse, representative group of beliefs regarding the flu vaccine and may have suggested additional areas for future directions, including community outreach and patient education for vaccine hesitancy. Further studies might include larger sample sizes across larger geographical regions to more comprehensively describe reasons for hesitancy regarding influenza vaccination in Hispanic individuals.

Another limitation includes possible user fatigue related to the length of our survey. Though only 23 questions in length with average time to completion of 5-10 minutes, the survey may have been too long, and it is possible that even amongst those who completed it (118 individuals, or 98% of the 120 who started the survey), the length may have affected their responses (hurried responses, incomplete comprehension, selective attention, etc.). While it is difficult to assess the veracity of these possibilities, future surveys may utilize fewer questions, fewer sections, and/or shorter prompts to decrease survey length and facilitate completion with full user comprehension. Our survey was primarily administered at public community locations and a community center during AMC events, allowing for a limited selection of survey respondents and possibly introducing selection bias. Our respondents often completed the survey through self-reported means, possibly introducing response bias. For instance, individuals could self-report and identify ethnicity, possibly introducing bias or subjectivity. However, this is a common limitation of survey-based data and describing demographics such as ethnicity, which are difficult to define and operationalize. We tried to mitigate any additional response bias by having investigators present to assist and perform interviewer administration of the survey when possible. Our study was also cross-sectional and non-randomized, meaning causality could not be demonstrated. Regardless of these limitations, we feel our study provided a meaningful snapshot of the local Hispanic community in Metro Detroit that future studies can build on.

Finally, as stated previously, our sample primarily included individuals with a history of influenza vaccination at least once in the past five years (93 respondents, 78% of the total). However, significant differences between this group of respondents and those without a history of vaccination in the same time period were few, mostly relating to trust (see Tables 13-17). We believe our study provided a meaningful introduction to factors influencing influenza vaccine behaviors and attitudes in the Hispanic community of Metro Detroit.

Future directions

Further studies will need to assess what other areas regarding vaccine purpose and function require additional investigation to improve patient knowledge and education, such as the misconception expressed above that the flu vaccine may give someone the flu. Recent studies [3,13,14,18] have suggested the usefulness of community-based efforts in addressing hesitancy and gaps in knowledge regarding vaccines, and such efforts may be uniquely implemented with the Hispanic community in the future. Further research regarding work trends and time-related barriers in the Metro Detroit and national Hispanic community is needed to further elucidate what non-health access-related barriers disproportionately affect Hispanic individuals and what measures can be taken to address these issues. Future studies might also include other methods for attracting more participants to recruit larger sample sizes, including options for virtual completion, incentives/compensation for participation, and additional promotion for informing the local community. Through collaboration with community partners, future research may reach more individuals and better determine what mistrust, if any, Hispanic residents have regarding vaccines and their reasons (such as whether government vaccination programs abroad and domestically generally lead to greater trust in vaccines). Future studies may also focus predominantly on Hispanic individuals in Metro Detroit and across the US who do not have significant previous vaccination history, so as to investigate variables related to vaccine hesitancy in this group.

## Conclusions

The Hispanic population in the United States of America is rapidly growing, and its health needs are dynamic and unique. While the growth in Michigan, and more specifically Metro Detroit, has trended upward, health data regarding the local Hispanic population is still quite scarce, especially related to important health behaviors such as influenza vaccination. Previous findings have suggested that the Hispanic population may experience limited knowledge, barriers, and a lack of trust regarding influenza vaccination. The data collected in our study suggests that knowledge, barriers to access, and trust regarding influenza vaccination are generally strong in the Metro Detroit Hispanic population. However, there may still be select gaps in vaccine knowledge, significant time-related barriers, and certain subgroups, such as Hispanic males and young individuals, may overall be less trusting of influenza vaccination. Efforts should be made through community initiatives to provide education and improve influenza vaccine knowledge, arrange time-flexible vaccination efforts, and perform targeted outreach to select subgroups with poor trust (such as young individuals and males) to increase influenza vaccination uptake for Hispanic individuals. More research is needed to investigate influenza vaccine knowledge, barriers, and trust in Hispanic individuals nationally and should be performed in neutral settings to increase generalizability and avoid health-related selection bias. With future research, additional community-based efforts may be implemented to improve influenza vaccine knowledge, barriers, and trust. Such efforts may have a considerable impact on vaccination rates and subsequent influenza outcomes of all Hispanic individuals.

## Appendices

Flu Vaccine Study (English)

Start of Block: Consent Form

Research Information Sheet Title of Study: Attitudes Towards Influenza Vaccine in Latinx Community of Southwest Detroit Principal Investigator: John Gallagher  Wayne State University School of Medicine Phone: (313) 241-0852 You are being asked to participate in a research survey about knowledge, barriers to access, and trust levels regarding flu vaccines and vaccines in general among community members in Southwest Detroit. This study is being conducted at the Ford Resource and Engagement Center/online via Qualtrics. Participation:  By completing the survey, you are agreeing to participate in this study. The data that you provide may be collected and used by Qualtrics as per its privacy agreement. Additionally, participation in this research is for residents of the United States over the age of 18; if you are not a resident of the United States and/or under the age of 18, please do not complete this survey. Study Procedures: If you take part in the study, you will be asked to fill out a survey that asks about your knowledge, barriers to access, and trust levels regarding flu vaccines and vaccines in general. No personal identifying information will be collected. You must answer all of the questions that are applicable to your situation to remain in the study. If you do not wish to continue with the survey, you can stop your participation at any time. This survey will take five minutes and will be completed during the Amigos Medicos Clinic/flu vaccination clinic. Benefits: As a participant in this research study, there will be no direct benefit for you; however, information from this study may indirectly benefit other people now or in the future. Risks: By taking part in this study, you may experience the following risks: possible loss of confidentiality. Costs: There will be no costs to you for participation in this research study. Compensation: You will not be paid for taking part in this study. Confidentiality: The survey will not collect any personal identifying information. All information collected about you during the course of this study will be kept without any identifiers. Voluntary Participation/Withdrawal: Taking part in this study is voluntary. You are free to not answer any questions or withdraw at any time. Your decision will not change any present or future relationships with Wayne State University, its affiliates, or Amigos Medicos Clinic. Questions: If you have any questions about this study now or in the future, you may contact John Gallagher or one of his research team members at the following phone number: (313) 241-0852. If you have questions or concerns about your rights as a research participant, the Chair of the Institutional Review Board can be contacted at (313) 577-1628. If you are unable to contact the research staff, or if you want to talk to someone other than the research staff, you may also call the Wayne State University Research Subject Advocate at (313) 577-1628 to discuss problems, obtain information, or offer comments.

End of Block: Consent Form

Start of Block: Flu Vaccine Study

The following survey intends to assess knowledge, barriers to access, and levels of trust regarding influenza (flu) vaccines in the Latinx (Latino/a/x) community of Southwest Detroit.

End of Block: Flu Vaccine Study

Start of Block: Demographics

Q1 What is your age?

o 18-24  (1)

o 25-34  (2)

o 35-44  (3)

o 45-54  (4)

o 55-64  (5)

o 65+  (6)

Q2 Please choose the race(s) that you identify with.

▢         American Indian or Alaska Native  (1)

▢         Asian  (2)

▢         Black or African American  (3)

▢         Native Hawaiian or Other Pacific Islander  (4)

▢         White  (5)

▢         Other  (6)

Q3 Do you identify as Hispanic or Latino/a?

o Yes  (1)

o No  (2)

Q4 Please select the gender you most identify with:

o Male  (1)

o Female  (2)

o Non-binary / third gender  (3)

o Prefer not to say  (4)

Q5 What is your preferred language?

o English  (1)

o Spanish  (2)

o Other  (3)

End of Block: Demographics

Start of Block: Previous Vaccine History

This portion of the survey asks about previous history related to vaccines and general opinions regarding vaccines.

Q6 How often in the past 5 years have you gotten a seasonal flu vaccine?

o Never  (1)

o Once  (2)

o A few times  (3)

o Every year  (4)

Q7 If your doctor recommends a vaccine, do you usually get it?

o Yes  (1)

o Sometimes  (2)

o No  (3)

Q8 Do you think vaccines in general are necessary?

o Yes  (1)

o Sometimes  (2)

o No  (3)

End of Block: Previous Vaccine History

Start of Block: Knowledge

Please indicate your level of agreement with the following statements:

Q9 The influenza (flu) vaccine may give me the flu.

o Strongly disagree  (1)

o Somewhat disagree  (2)

o Neither agree nor disagree  (3)

o Somewhat agree  (4)

o Strongly agree  (5)

Q10 The influenza (flu) vaccine is safe for pregnant women.

o Strongly disagree  (1)

o Somewhat disagree  (2)

o Neither agree nor disagree  (3)

o Somewhat agree  (4)

o Strongly agree  (5)

Q11 If I got the influenza (flu) vaccine last year, I don't need to get it again this year.

o Strongly disagree  (1)

o Somewhat disagree  (2)

o Neither agree nor disagree  (3)

o Somewhat agree  (4)

o Strongly agree  (5)

Q12 If I am healthy, I don't need the influenza (flu) vaccine.

o Strongly disagree  (1)

o Somewhat disagree  (2)

o Neither agree nor disagree  (3)

o Somewhat agree  (4)

o Strongly agree  (5)

Q13 The influenza (flu) vaccine may interact with medications.

o Strongly disagree  (1)

o Somewhat disagree  (2)

o Neither agree nor disagree  (3)

o Somewhat agree  (4)

o Strongly agree  (5)

End of Block: Knowledge

Start of Block: Barriers to Access

Please indicate your level of agreement with the following statements:

Q14 I need medical insurance to get the influenza (flu) vaccine.

o Strongly disagree  (1)

o Somewhat disagree  (2)

o Neither agree nor disagree  (3)

o Somewhat agree  (4)

o Strongly agree  (5)

Q15 It would be difficult for me to cover the cost required for an influenza (flu) vaccine.

o Strongly disagree  (1)

o Somewhat disagree  (2)

o Neither agree nor disagree  (3)

o Somewhat agree  (4)

o Strongly agree  (5)

Q16 It would be difficult for me to find transportation to make an influenza (flu) vaccination appointment.

o Strongly disagree  (1)

o Somewhat disagree  (2)

o Neither agree nor disagree  (3)

o Somewhat agree  (4)

o Strongly agree  (5)

Q17 I would not know where to get the influenza (flu) vaccine if I wanted one.

o Strongly disagree  (1)

o Somewhat disagree  (2)

o Neither agree nor disagree  (3)

o Somewhat agree  (4)

o Strongly agree  (5)

Q18 Please indicate if you may experience any of the following barriers to getting the influenza (flu) vaccine:

▢         Against my beliefs  (1)

▢         Lack of access/availability  (2)

▢         Lack of insurance  (3)

▢         Lack of time  (4)

▢         Lack of transportation  (5)

▢         Medical/allergic condition  (6)

▢         Too expensive  (7)

▢         Other  (8) __________________________________________________

End of Block: Barriers to Access

Start of Block: Level of Trust

Please indicate your level of agreement with the following statements:

Q19 The influenza (flu) vaccine prevents people from getting sick.

o Strongly disagree  (1)

o Somewhat disagree  (2)

o Neither agree nor disagree  (3)

o Somewhat agree  (4)

o Strongly agree  (5)

Q20 Government influenza (flu) vaccination programs are trustworthy.

o Strongly disagree  (1)

o Somewhat disagree  (2)

o Neither agree nor disagree  (3)

o Somewhat agree  (4)

o Strongly agree  (5)

Q21 The influenza (flu) vaccine manufacturers are trustworthy.

o Strongly disagree  (1)

o Somewhat disagree  (2)

o Neither agree nor disagree  (3)

o Somewhat agree  (4)

o Strongly agree  (5)

Q22 The ingredients in the influenza (flu) vaccines are generally non-toxic.

o Strongly disagree  (1)

o Somewhat disagree  (2)

o Neither agree nor disagree  (3)

o Somewhat agree  (4)

o Strongly agree  (5)

End of Block: Level of Trust

Start of Block: Reasons for Vaccine Refusal

Q23 If you are not willing to receive the influenza (flu) vaccine please indicate why:

________________________________________________________________

End of Block: Reasons for Vaccine Refusal

Flu Vaccine Study (Spanish)

Start of Block: Consent Form

Hoja de información de investigación  Título del estudio: Actitudes sobre la vacuna contra la influenza en la comunidad latinx del suroeste de Detroit  Investigador principal: John Gallagher  Escuela de Medicina de Wayne State University Teléfono: (313) 241-0852  Se le pide que participe en una encuesta de investigación sobre el conocimiento, las barreras de acceso y los niveles de confianza con respecto a las vacunas contra la influenza y las vacunas en general entre los miembros de la comunidad en el suroeste de Detroit. Este estudio se lleva a cabo en el Ford Resource and Engagement Center/en línea a través de Qualtrics.     Participación: Al completar la encuesta, acepta participar en este estudio. Los datos que proporcione pueden ser recopilados y utilizados por Qualtrics según su acuerdo de privacidad. Además, la participación en esta investigación es para residentes de los Estados Unidos mayores de 18 años; si no es residente de los Estados Unidos y/o tiene menos de 18 años, no complete esta encuesta. Procedimientos de estudio: Si participa en el estudio, se le pedirá que complete una encuesta sobre su conocimiento, las barreras de acceso y los niveles de confianza con respecto a las vacunas contra la influenza y las vacunas en general. Ninguna informacion de identificación personal será recopilada. Debe responder todas las preguntas que correspondan a su situación para permanecer en el estudio. Si no desea continuar con la encuesta, puede terminar su participación en cualquier momento. Esta encuesta tomará cinco minutos y se completará durante la jornada de salud de Clínica Amigos Médicos/día de vacunación.     Beneficios:  Como participante en este estudio de investigación, no habrá ningún beneficio directo para usted; sin embargo, la información de este estudio puede beneficiar indirectamente a otras personas ahora o en el futuro. Riesgos: Al participar en este estudio, puede experimentar los siguientes riesgos: posible pérdida de confidencialidad. Costos: No habrá costos para usted por participar en este estudio de investigación. Compensación:  No se le pagará por participar en este estudio. Confidencialidad:  La encuesta no recopilará ninguna información de identificación personal. Toda la información recopilada sobre usted durante el curso de este estudio se mantendrá sin identificadores. Participación/Retiro Voluntario:  La participación en este estudio es voluntaria. Usted es libre de no responder ninguna pregunta o retirarse en cualquier momento. Su decisión no cambiará ninguna relación presente o futura con Wayne State University, sus afiliados o la Clínica Amigos Médicos. Preguntas: Si tiene alguna pregunta sobre este estudio ahora o en el futuro, puede comunicarse con John Gallagher o con uno de los miembros de su equipo de investigación al siguiente número de teléfono: (313) 241-0852. Si tiene preguntas o inquietudes sobre sus derechos como participante de la investigación, puede comunicarse con el Presidente de la Junta de Revisión Institucional al (313) 577-1628. Si no puede comunicarse con el personal de investigación, o si desea hablar con alguien que no sea el personal de investigación, también puede llamar al Defensor de Sujetos de Investigación de Wayne State University al (313) 577-1628 para discutir problemas, obtener información u ofrecer comentarios.

End of Block: Consent Form

Start of Block: Flu Vaccine Study

La siguiente encuesta pretende evaluar el conocimiento, las barreras de acceso y los niveles de confianza con respecto a las vacunas contra la influenza (gripe) en la comunidad latina del suroeste de Detroit

End of Block: Flu Vaccine Study

Start of Block: Demographics

Q1 ¿Cuál es su edad?

o 18-24  (1)

o 25-34  (2)

o 35-44  (3)

o 45-54  (4)

o 55-64  (5)

o 65+  (6)

Q2 Elija la(s) raza(s) con las que se identifica.

▢         Indio americano o nativo de Alaska  (1)

▢         Asiático  (2)

▢         Negro o afroamericano  (3)

▢         Nativo de Hawai u otra isla del Pacífico  (4)

▢         Blanco  (5)

▢         Otro  (6)

Q3 ¿Se identifica como hispano o latino?

o Sí  (1)

o No  (2)

Q4 Seleccione el género con el que más se identifica:

o Masculino  (1)

o Femenino  (2)

o No binario / tercer género  (3)

o Prefiero no decirlo  (4)

Q5 ¿Cuál es su idioma preferido?

o Inglés  (1)

o Español  (2)

o Otro  (3)

End of Block: Demographics

Start of Block: Previous Vaccine History

Esta parte de la encuesta pregunta sobre el historial previo relacionado con las vacunas y las opiniones generales sobre las vacunas.

Q6 ¿Con qué frecuencia en los últimos 5 años se ha vacunado contra la gripe estacional?

o Nunca  (1)

o Una vez  (2)

o Unas pocas veces  (3)

o Todos los años  (4)

Q7 Si su médico le recomienda una vacuna, ¿usualmente la recibe?

o Sí  (1)

o A veces  (2)

o No  (3)

Q8 ¿Cree que las vacunas en general son necesarias?

o Sí  (1)

o A veces  (2)

o No  (3)

End of Block: Previous Vaccine History

Start of Block: Knowledge

Indique su nivel de acuerdo con las siguientes afirmaciones:

Q9 La vacuna contra la influenza (gripe) puede darme gripe.

o Muy en desacuerdo  (1)

o Algo en desacuerdo  (2)

o Ni de acuerdo ni en desacuerdo  (3)

o Parcialmente de acuerdo  (4)

o Totalmente de acuerdo  (5)

Q10 La vacuna contra la influenza (gripe) es segura para las mujeres embarazadas.

o Muy en desacuerdo  (1)

o Algo en desacuerdo  (2)

o Ni de acuerdo ni en desacuerdo  (3)

o Parcialmente de acuerdo  (4)

o Totalmente de acuerdo  (5)

Q11 Si me vacuné contra la influenza (gripe) el año pasado, no necesito vacunarme nuevamente este año.

o Muy en desacuerdo  (1)

o Algo en desacuerdo  (2)

o Ni de acuerdo ni en desacuerdo  (3)

o Parcialmente de acuerdo  (4)

o Totalmente de acuerdo  (5)

Q12 Si estoy sano/a, no necesito la vacuna contra la influenza (gripe).

o Muy en desacuerdo  (1)

o Algo en desacuerdo  (2)

o Ni de acuerdo ni en desacuerdo  (3)

o Parcialmente de acuerdo  (4)

o Totalmente de acuerdo  (5)

Q13 La vacuna contra la influenza (gripe) puede interactuar con los medicamentos.

o Muy en desacuerdo  (1)

o Algo en desacuerdo  (2)

o Ni de acuerdo ni en desacuerdo  (3)

o Parcialmente de acuerdo  (4)

o Totalmente de acuerdo  (5)

End of Block: Knowledge

Start of Block: Barriers to Access

Indique su nivel de acuerdo con las siguientes afirmaciones:

Q14 Necesito un seguro médico para recibir la vacuna contra la influenza (gripe).

o Muy en desacuerdo  (1)

o Algo en desacuerdo  (2)

o Ni de acuerdo ni en desacuerdo  (3)

o Parcialmente de acuerdo  (4)

o Totalmente de acuerdo  (5)

Q15 Sería difícil para mí cubrir el costo requerido para una vacuna contra la influenza (gripe).

o Muy en desacuerdo  (1)

o Algo en desacuerdo  (2)

o Ni de acuerdo ni en desacuerdo  (3)

o Parcialmente de acuerdo  (4)

o Totalmente de acuerdo  (5)

Q16 Sería difícil para mí encontrar transporte para programar una cita de vacunación contra la influenza (gripe).

o Muy en desacuerdo  (1)

o Algo en desacuerdo  (2)

o Ni de acuerdo ni en desacuerdo  (3)

o Parcialmente de acuerdo  (4)

o Totalmente de acuerdo  (5)

Q17 No sabría dónde obtener la vacuna contra la influenza (gripe) si quisiera una.

o Muy en desacuerdo  (1)

o Algo en desacuerdo  (2)

o Ni de acuerdo ni en desacuerdo  (3)

o Parcialmente de acuerdo  (4)

o Totalmente de acuerdo  (5)

Q18 Por favor indique si tiene o ha tenido alguna de las siguientes barreras para recibir la vacuna contra la influenza (gripe):

▢         Contra mis creencias  (1)

▢         Falta de acceso/disponibilidad  (2)

▢         falta de seguro  (3)

▢         Falta de tiempo  (4)

▢         falta de transporte  (5)

▢         Condición médica/alérgica  (6)

▢         Muy caro  (7)

▢         Otro  (8) __________________________________________________

End of Block: Barriers to Access

Start of Block: Level of Trust

Indique su nivel de acuerdo con las siguientes afirmaciones:

Q19 Las vacunas contra la influenza (gripe) evitan que las personas se enfermen.

o Muy en desacuerdo  (1)

o Algo en desacuerdo  (2)

o Ni de acuerdo ni en desacuerdo  (3)

o Parcialmente de acuerdo  (4)

o Totalmente de acuerdo  (5)

Q20 Los programas gubernamentales de vacunación contra la influenza (gripe) son confiables.

o Muy en desacuerdo  (1)

o Algo en desacuerdo  (2)

o Ni de acuerdo ni en desacuerdo  (3)

o Parcialmente de acuerdo  (4)

o Totalmente de acuerdo  (5)

Q21 Los fabricantes de vacunas contra la influenza (gripe) son confiables.

o Muy en desacuerdo  (1)

o Algo en desacuerdo  (2)

o Ni de acuerdo ni en desacuerdo  (3)

o Parcialmente de acuerdo  (4)

o Totalmente de acuerdo  (5)

Q22 Los ingredientes de las vacunas contra la influenza (gripe) generalmente no son tóxicos.

o Muy en desacuerdo  (1)

o Algo en desacuerdo  (2)

o Ni de acuerdo ni en desacuerdo  (3)

o Parcialmente de acuerdo  (4)

o Totalmente de acuerdo  (5)

End of Block: Level of Trust

Start of Block: Reasons for Vaccine Refusal

Q23 Si no está dispuesto/a a recibir la vacuna contra la influenza (gripe), indique por qué:

________________________________________________________________

End of Block: Reasons for Vaccine Refusal
